# Combined Volumetric and Density Analyses of Contrast-Enhanced CT Imaging to Assess Drug Therapy Response in Gastroenteropancreatic Neuroendocrine Diffuse Liver Metastasis

**DOI:** 10.1155/2018/6037273

**Published:** 2018-10-25

**Authors:** Yi Wang, Kun Huang, Jie Chen, Yanji Luo, Yu Zhang, Yingmei Jia, Ling Xu, Minhu Chen, Bingsheng Huang, Dong Ni, Zi-Ping Li, Shi-Ting Feng

**Affiliations:** ^1^National-Regional Key Technology Engineering Laboratory for Medical Ultrasound, Guangdong Key Laboratory for Biomedical Measurements and Ultrasound Imaging, School of Biomedical Engineering, Health Science Center, Shenzhen University, Shenzhen, China; ^2^Department of Radiology, The First Affiliated Hospital, Sun Yat-Sen University, Guangzhou, China; ^3^Department of Gastroenterology, The First Affiliated Hospital, Sun Yat-Sen University, Guangzhou, China; ^4^Faculty of Medicine and Dentistry, University of Western Australia, Perth 6009, Australia

## Abstract

**Objective:**

We propose a computer-aided method to assess response to drug treatment, using CT imaging-based volumetric and density measures in patients with gastroenteropancreatic neuroendocrine tumors (GEP-NETs) and diffuse liver metastases.

**Methods:**

Twenty-five patients with GEP-NETs with diffuse liver metastases were enrolled. Pre- and posttreatment CT examinations were retrospectively analyzed. Total tumor volume (volume) and mean volumetric tumor density (density) were calculated based on tumor segmentation on CT images. The maximum axial diameter (tumor size) for each target tumor was measured on pre- and posttreatment CT images according to Response Evaluation Criteria In Solid Tumors (RECIST). Progression-free survival (PFS) for each patient was measured and recorded.

**Results:**

Correlation analysis showed inverse correlation between change of volume and density (Δ(*V* + *D*)), change of volume (Δ*V*), and change of tumor size (Δ*S*) with PFS (*r* = −0.653, *P*=0.001; *r* = −0.617, *P*=0.003; *r* = −0.548, *P*=0.01, respectively). There was no linear correlation between Δ*D* and PFS (*r* = −0.226, *P*=0.325).

**Conclusion:**

The changes of volume and density derived from CT images of all lesions showed a good correlation with PFS and may help assess treatment response.

## 1. Introduction

Neuroendocrine tumors (NETs) may affect different organs of the human body, such as the thymus, lungs, pancreas, and gastrointestinal tract [1, 2]. The estimated annual incidence of clinically significant NETs is 5.25 per 100,000 [3]. Gastroenteropancreatic (GEP) neuroendocrine tumors (GEP-NETs) are the most common NETs, accounting for 65–75% of all NETs [4, 5].

Liver is the most common site for GEP-NETs metastases; between 65 and 95% of GEP-NETs metastasize to the liver [6–8]. Diffuse liver metastases occur in most patients with GEP-NETs and directly influence prognosis in patients. Many treatment strategies can be applied to GEP-NETs with diffuse liver metastasis, of which medications play an important role in the management of unresectable liver metastases. Current standard medical treatment options include the use of somatostatin analogues, cytotoxic chemotherapy agents, and targeted agents [8].

Tumor treatment response can provide prognostic information and assist in determining the follow-up treatment strategy. Several metrics have been used in monitoring tumor response, among which chromogranin A (CgA) is currently recognized as the most valuable serum tumor marker used for screening, diagnosis, treatment, monitoring of progress, and prognostic evaluation [9–12]. However, there is no universal detection method, and the normal range and sensitivity of CgA vary according to the assay used [9–12]. This limitation restricts its application in current clinical practice.

The use of computed tomography (CT) images is one of the most versatile and intuitive method [13]. RECIST [14] is widely used for the evaluation of treatment outcomes of anticancer therapy. However, RECIST criteria rely on diameter measurements on a single cross-sectional plane and on a limited number of lesions, making its interpretation controversial [15–18]. While some studies support its use for evaluating tumor response to treatment [15], others have suggested that it may overestimate or underestimate tumor burden [16–18]. Recent reports have claimed superiority of volumetric measurement of tumors over the use of RECIST [18–20]. However, a majority of these studies involved several representative lesions and did not reflect the change in all detectable hepatic lesions in patients with diffuse liver metastasis [18–20].

This study proposed a new computer-aided method to assess the treatment response using changes in total tumor volume and mean volumetric tumor density derived from volumetric measurements of all lesions in patients with GEP-NET diffuse metastases treated with drug therapy. The objective of this study was to assess the correlation of the changes in volumetric metrics with PFS that reflected the therapeutic effect. An analysis of its advantages and disadvantages over RECIST criteria is presented.

## 2. Materials and Methods

### 2.1. Patient Population

The study protocol was reviewed and approved by the Ethics Committee of Sun Yat-Sen University, and informed consent was obtained from all patients. The methods were carried out in accordance with the approved guidelines. A total of 45 patients with GEP-NET diffuse liver metastases, who received at least two courses of drug therapy after resection of the primary tumor between February 2012 and June 2015, were retrospectively collected for this study. Subsequently, further screening was performed according to the inclusion criteria. Inclusion criteria were: availability of pre- and posttreatment dual-phase contrast-enhanced spiral CT images and progression-free survival (PFS), and absence of extrahepatic metastases. Twenty patients were excluded from the study due to either lack of data on posttreatment PFS (*N* = 4), lack of either pre- or posttreatment CT images (*N* = 10), or due to other extrahepatic disease that affected treatment outcomes (*N* = 5); one patient had unqualified CT images. Finally, a total of 25 patients (18 males and 7 females) came within the purview of this retrospective analysis, of which 10 cases had primary tumors located in the pancreas, 3 cases were located in the stomach, 7 cases were located in the small bowel, and 5 cases were located in the rectum (Table 1).

### 2.2. Treatment Protocol

The drug therapy protocol was followed systematically. All 25 patients had received at least two courses of treatment (7 patients had two courses; 6 patients had three courses; 6 patients had four courses; 2 patients had five courses; 3 patients had six courses; and 1 patient had eight courses). Five treatment protocols were used in patients included in this study. Six patients received protocol (1) sunitinib (37.5 mg orally, qd). Three patients received protocol (2) everolimus (5 mg, orally. qd). One patient received protocol (3) capecitabine and temozolomide (capecitabine: 1.0 g orally, after breakfast, 1.5 g orally, after supper, d1-d14; temozolomide: 300 mg orally, half an hour before lunch, d10-d14, q4w). Seven patients received protocol (4) etoposide and cisplatin (etoposide: 100 mg intravenous. qd, d1-d5; cisplatin: 30 mg intravenous. qd, d1-d4, q3w). Eight patients received protocol (5) octreotide acetate (30 mg intramuscular injection, q4w).

### 2.3. CT Imaging Protocol

All patients underwent pre- and posttreatment CT imaging according to the standard institutional protocol for imaging of gastroenteropancreatic neuroendocrine liver metastasis. The posttreatment CT was performed after the end of two courses of treatment. A 64-row spiral CT (Toshiba Aquilion64, Japan) equipment was used. Breath-hold unenhanced and contrast-enhanced images (matrix, 512 × 512; slice thickness, 1 mm; interslice gap, 0.8 mm) were obtained in the arterial (37 s) and portal venous phases (65 s). For contrast-enhanced CT, a dose of 1.5 ml/kg iopromide (Ultravist300, Schering, Berlin, Germany) was administrated at a rate of 3–4 ml/s. CT scan was obtained before contrast agent injection, 34–37 s and 60–70 s after contrast agent injection, respectively.

### 2.4. RECIST Measurement

The follow-up observation was performed according to RECIST 1.1, which is based on the evaluation of a maximum of two target lesions per organ [21]. The sum of the longest diameters of the target lesions in each patient was computed in portal phase. Then, the percentage change in tumor size from pretreatment levels was computed for each patient. The following were the definitions of the response criteria. Complete response (CR): disappearance of all target lesions. Partial response (PR): at least a 30% decrease in the sum of diameters of target lesions, taking as reference the baseline sum diameters. Progressive disease (PD): at least a 20% increase and an absolute increase of at least 5 mm in the sum of diameters of target lesions. Stable disease (SD) was defined as neither sufficient shrinkage to qualify for PR nor sufficient increase to qualify for PD. And, all patients were divided into two groups: progressive tumor (PD) and nonprogression tumor (PR or SD).

### 2.5. Progression-Free Survival (PFS)

Progression-free survival (PFS) is defined as the time between treatment initiation and evidence of tumor progression or death from any cause, with censoring of patients who are lost to follow-up or no tumor progression.

### 2.6. Volumetric Measurements

**Figure 1:**
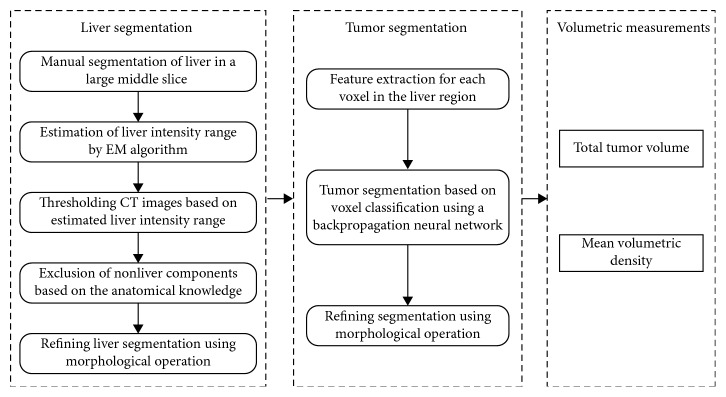
Schematic diagram of the proposed volumetric measurements.

In this study, a neural network classifier [22–24] was used to segment tumors from 3D CT liver images in the portal phase. Specifically, our whole segmentation framework consisted of two components (Figure 1): (1) liver segmentation and (2) tumor segmentation from liver region. We adopted [22] to conduct liver segmentation, and combined [23] and [24] to segment tumor from liver region. In the first step, the liver region was identified by threshold method based on intensity analysis and anatomical knowledge [22]. We formulated the intensity distribution of CT images as a Gaussian mixture model with two components that represented liver region and nonliver regions, respectively. A large middle slice of liver was firstly segmented by a CT radiologist manually. Then, the intensity range of the liver region was estimated by analyzing the statistical parameters of the Gaussian mixture model using expectation-maximization algorithm in this slice. The estimated intensity range was then used to threshold the images for liver segmentation. In order to discard components with a similar intensity as that of liver, we further refined the liver segmentation by keeping the largest connective region while discarding other regions based on the anatomical knowledge. And a morphological closing operation was performed to remove small holes in the segmented liver region.

Then, in the second step, within the segmented liver region, a backpropagation neural network-based classification method was employed to segment the tumors based on a series of grayscale co-occurrence features and statistical features [23, 24]. The backpropagation neural network consisted of four layers, that is, one input layer, two hidden layers, and one output layer, which was trained using eight sets of CT liver images. For each voxel in the liver region, we extracted the statistical features and co-occurrence matrix-based features (e.g., average intensity, entropy, contrast, correlation [23]) within its 11 × 11 × 11 neighborhood. These features were then fed to the backpropagation neural network for classification of the voxel as tumor or nontumor. Then, morphological closing and opening operations were performed to remove small holes and small noises, respectively. Finally, the tumor segmentation results were further refined by a CT radiologist.

The following metrics were calculated based on the segmentation results: (1) total tumor volume: the sum of the tumor volumes from each segmented tumor was calculated for each patient, and the absolute and percent change in the sum from pretreatment levels computed; (2) mean volumetric tumor density: the mean CT value of all the tumor tissues as well as the absolute and percent change from pretreatment level was calculated for each patient.

### 2.7. Statistical Analysis

Tumor volume change (Δ*V*), tumor attenuation change (Δ*D*), and tumor size change (Δ*S*) were defined as the difference of volume, density, and size between CT pre- and posttreatment, respectively. The combined tumor volume change and tumor attenuation change (Δ(*V* + *D*)) was calculated by addition. For example, if the tumor density decreases by 4.7% and the tumor volume increases by 131.25% (patient 12), this results in a Δ(*V* + *D*) of 126.55% (positive value). The data of Δ*V*, Δ*D*, Δ*S*, and Δ(*V* + *D*) on each patient are shown in Table 2.

To investigate the value of total tumor volume and mean volumetric tumor density independently or in combination with treatment response, correlation analysis was performed. Correlation of Δ(*V* + *D*), Δ*V*, Δ*D*, and Δ*S* with PFS was assessed by Pearson correlation (*r*). Statistical analysis was performed using SPSS 20.0 software. *P*<0.05 was considered statistically significant.

## 3. Results

The number of hepatic lesions in an individual patient ranged from 6 to 129 before treatment and from 11 to 135 after treatment. The sum of the longest diameters of the target lesions of an individual patient according to RECIST criteria ranged from 1.4 to 19.5 cm (pretreatment) and from 2.0 to 23.5 cm (posttreatment). The total tumor volume of an individual patient ranged from 0.8 to 2168.0 cm^3^ before treatment and from 1.7 to 2707.0 cm^3^ after treatment. The mean volumetric tumor density in an individual patient ranged from 55.7 to 126.1 Hounsfield unit (HU) before treatment and from 50.7 to 143.5 HU after treatment.

**Figure 2:**
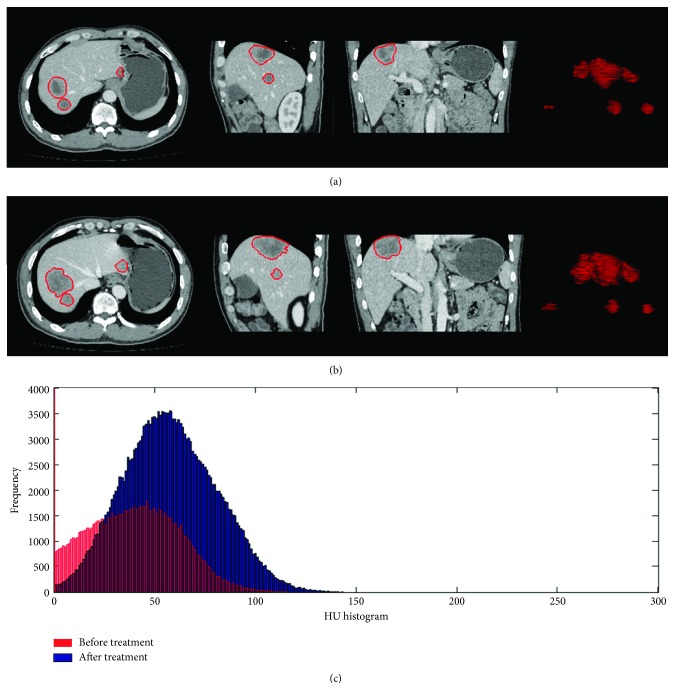
The tumor volume and HU histogram changes in one of the cases. Gastroenteropancreatic neuroendocrine liver metastasis pre- and posttreatment illustrating total tumor volume and mean volumetric tumor density changes from contrast-enhanced CT images in a patient with tumor regression. GEP-NETs with liver metastasis pretreatment (a) show a pretreatment tumor volume of 57.4 cm^3^, whereas in (b) the posttreatment, tumor volume was 40.9 cm^3^. The HU histogram (c) shows a left side shift, representing decreased enhancement in the posttreatment CT imaging, that is, HU value decreased from 80.2 to 68.1 HU.

**Figure 3:**
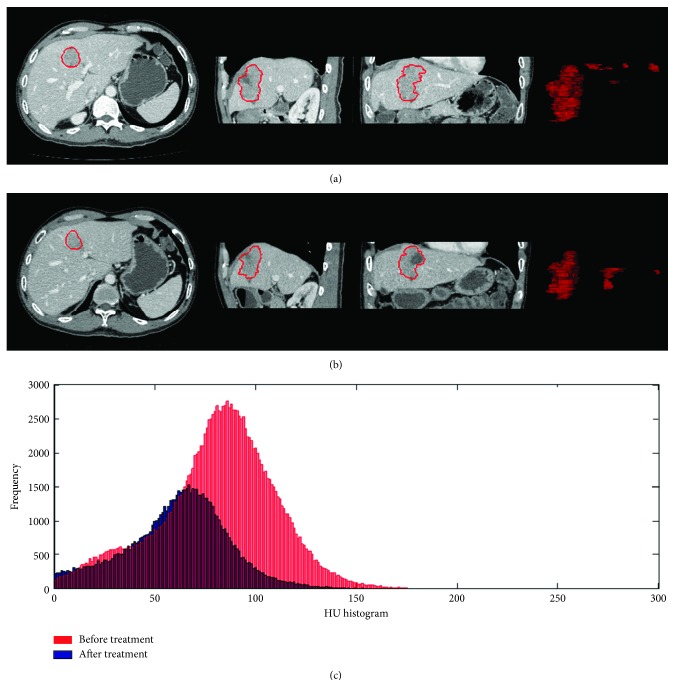
The tumor volume and HU histogram changes in one of the cases. Gastroenteropancreatic neuroendocrine liver metastasis pre- and posttreatment illustrating total tumor volume and mean volumetric tumor density changes from contrast-enhanced CT images in a single tumor progression patient. GEP-NETs with liver metastasis pretreatment (a) show a pretreatment tumor volume of 45.1 cm^3^, whereas in (b) the posttreatment, tumor volume was 77.6 cm^3^. The HU histogram (c) shows a rightward shift, representing increased enhancement on posttreatment CT imaging. The tumor HU value increased from 45.7 to 57.8 HU.

According to the changes of the maximum axial diameter before and after treatment, 7 patients showed evidence of tumor progression, while the other 18 patients showed nonprogression (4 partial response and 14 with stable disease). In the tumor progression group, the tumor size increased by a mean of 175% after treatment. In contrast, the tumor size decreased by a mean of 13.5% after treatment in the tumor nonprogression group. For the tumor progression group, the mean increase in total tumor volume was 134.1%, but the mean decrease in volumetric tumor density was 8.7%. For the tumor nonprogression group, the total tumor volume increased by a mean of 36.6%, but the mean volumetric tumor density increased by a mean of 6.4%. Figures 2 and 3 show the tumor volume and HU histogram changes before and after treatment in one case of tumor regression and one case of tumor progression. Supplement video file 1 shows a 3D visualization of the segmented tumors in one patient.

Changes in total tumor volume and mean volumetric tumor density pre- and posttreatment for all 25 patients after treatment are shown in Tables 2 and 3. The total tumor volume and mean volumetric tumor density showed opposite changes in 6 patients (Table 3). Of 7 patients with tumor progression according to RECIST 1.1, 3 patients demonstrated increased total tumor volume and mean volumetric tumor density: the total tumor volume increased by 287.3%, 31.8%, and 81.3%, respectively, and the mean volumetric tumor density increased by 1.7%, 17.1%, and 11.0%, respectively. In another 3 patients with increased total tumor volumes and decreased mean volumetric tumor density, the total tumor volume increased by 296%, 131.2%, and 112.5%, respectively, while the mean volumetric tumor density decreased by 31.7%, 4.7%, and 24.2%, respectively. The remaining 1 patient demonstrated a decrease in both total tumor volume and mean volumetric tumor density by 1.6% and 30.2%, respectively. Of 18 patients with stable disease, 2 patients demonstrated decreased total tumor volume and mean volumetric tumor density: the total tumor volume decreased by 88.7% and 74.6%, and the mean volumetric tumor density decreased by 3.0% and 27.8%, respectively. 2 patients demonstrated increased total tumor volume and decreased mean volumetric tumor density, the total tumor volume increased by 57.9% and 43.5%, and the mean volumetric tumor density decreased by 14.5% and 13.1%, respectively. 2 patients with decreased total tumor volume and increased mean volumetric tumor density, the total tumor volume decreased by 28.7% and 27.5%, and the mean volumetric tumor density increased by 17.8% and 5.4%, respectively. 12 showed increased total tumor volume and mean volumetric tumor density.

During the study period, tumor progression was observed in 21 patients (84%). The remaining 4 patients (16%) exhibited no evidence of tumor progression. The median PFS of these 21 patients was 4.1 months (range 1.8–9.4 months).

**Figure 4:**
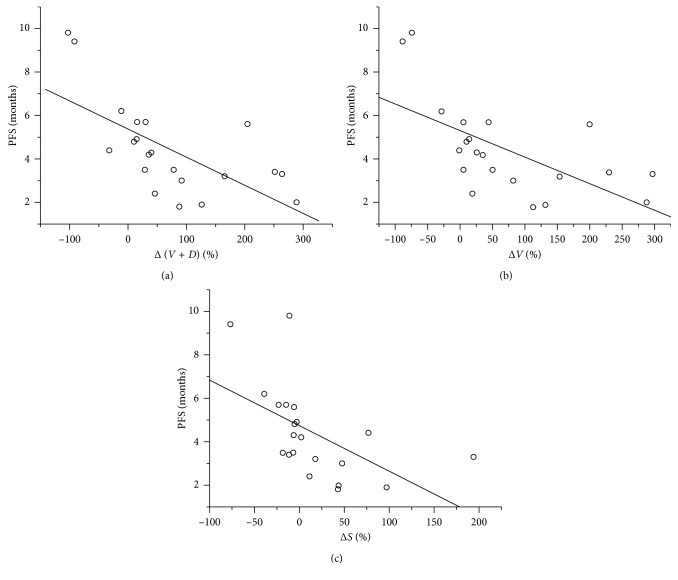
Scatterplots show inverse correlation of (a) change of volume and density (Δ(*V* + *D*)), (b) change of volume (Δ*V*), and (c) change of tumor size (Δ*S*) with PFS (*r* = −0.653, *P*=0.001; *r* = −0.617, *P*=0.003; *r* = −0.548, *P*=0.01).

On correlation analysis (Figure 4), there was inverse linear correlation between Δ(*V* + *D*) and PFS (*r* = −0.653, *P*=0.001), Δ*V* and PFS (*r* = −0.617, *P*=0.003), and Δ*S* according to RECIST and PFS (*r* = −0.548, *P*=0.01). No linear correlation was found between Δ*D* and PFS (*r* = −0.226, *P*=0.325). The inverse linear correlation between Δ(*V* + *D*) and PFS (*r* = −0.653) was higher than that of Δ*S* according to RECIST and PFS (*r* = −0.548).

## 4. Discussion

Our results demonstrated that the total tumor volume and mean tumor density obtained using volumetric measurements correlated better with PFS than tumor size of RECIST 1.1. Similar results have been reported elsewhere [18, 25]. Hayes et al. reported superiority of volumetric measurements over use of unidimensional RECIST criteria to predict overall survival [25]. Welsh et al. also suggested volumetric analysis as the preferred method to detect tumor progression [18].

Just as some of the previous studies reported that RECIST might significantly underestimate or overestimate the tumor burden [16, 18], our research suggested RECIST criteria were inadequate for a precise assessment of the tumor treatment response [17]. Being a unidimensional tumor metric, RECIST criteria were a valid surrogate for three-dimensional growth in tumors, only in the case of spherical-shaped tumors. However, liver metastases tend to have complicated shapes where it could not be accurately represented using RECIST metric.

Medications do not always cause tumoral shrinkage. Treatment response observed with targeted therapies could manifest as decrease in lesion size, decrease in lesion vascularity, cystic changes, and intratumoral hemorrhage, with or without a change in size [26]. This theory might also apply to nontargeted drugs. Reduction in tumor size is usually minimal during the early stages of treatment despite significant changes such as a decrease in the number of vessels [27]. Therefore, morphological response assessment may not always provide an objective measure.

Functional imaging is increasingly being used for monitoring of response to anticancer therapy. CT enhancement correlates with tumor vascularity; so, the attenuation might indirectly reflect tumor activity. Several investigators had previously demonstrated that assessment of tumor density could improve therapeutic response assessment in metastatic gastrointestinal tumors [28, 29]. However, at present, most of the studies have just measured the tumor CT density of different representative layers. Attenuation of selected level does not reflect the overall treatment response well. There is a growing recognition that intratumor heterogeneity could affect therapeutic effects in different areas of the same tumor [30].

Volumetric measurements of tumor response would reflect comprehensively the therapeutic effects of the various parts. Vargas et al. found a significant association between tumor grade and enhancement only when measuring the entire tumor and not on the most enhanced portion on a single slice [31]. Evaluation of whole-lesion attenuation had shown a better reproducibility than that of region of interest (ROI) measurements [32]. We measured the pre- and posttreatment mean volumetric density of the entire tumor and found no significant association between tumor density and PFS. However, combined tumor volume and density has been shown to better reflect therapeutic response. Smith et al. also found that evaluating changes in both tumor size and tumor density after targeted therapy remarkably improved the response assessment in metastatic renal cell carcinoma [33]. Our research demonstrated higher inverse linear correlation when combining change of total tumor volume and mean volumetric tumor density than using any one of these alone. This was also observed when compared with the change in tumor size measured using RECIST and PFS. For example, tumor necrosis might result in increase in volume and a concomitant decrease in density. Therapeutic effect depends on the change in the amounts and activity of tumor tissue. Therefore, an analysis of the degree of change in total tumor volume and mean volumetric tumor density is more meaningful for purposes of treatment evaluation. The actual treatment response could be comprehensively determined using the percentage change in both total tumor volume and the mean volumetric tumor density.

Development of postprocessing imaging methods has allowed three-dimensional assessment of lesion segmentation and volumetry. To overcome the limitations of traditional tumor response criteria, we proposed a computer-aided method to calculate volume and volumetric density. Unlike the methods reported in the literature, we aimed to calculate the volume and density of all the lesions. The use of RECIST involves selection of several representative lesions and does not consider lesions <1.0 cm. In most studies, a limited number of lesions were considered representative of all lesions for calculation of tumor volume. Further, several representative layers are chosen for calculation of tumor density. However, owing to heterogeneity among the lesions, a limited sample of lesions is not necessarily representative of the whole [30]. Our study included all lesions (even <1.0 cm) and some patients had more than 100 lesions. The proposed method can be easily generalized to other segmentation applications. With the wide use of medical therapy for various tumors, precise assessment of therapy response becomes more and more challenged. Our method might be improved to make it applicable to various usages in clinical imaging assessment for multiple metastases of different solid tumors, for example, lung cancer, breast tumor, and lymphoma. Furthermore, the popular deep learning methods could also be employed for more accurate and efficient organ and tumor segmentation in our future work.

There were several limitations of our study. Firstly, due to the low overall incidence of GEP-NETs, there was a mixture of NET types with the primary in the pancreas, stomach, small intestine, and rectum, which may make it difficult to draw firm conclusions in heterogeneous patient group. Secondly, the sample size of our study is relatively small; to further study the superiority of volume and density measurement of tumors, a larger sample size is needed. Thirdly, the several different treatments applied also add to this problem of drawing firm conclusions from the present results.

## 5. Conclusions

We combined total volumetric and density analyses of contrast-enhanced CT imaging for assessment of therapeutic response in patients with GEP-NETs with diffuse liver metastasis. The volumetric and density analyses were carried out using semiautomatic segmentation of all tridimensional metastases. The combined use of total tumor volume and mean volumetric tumor density derived from the volumetric measurement may be helpful in the assessment of treatment response in patients with GEP-NETs with diffuse liver metastasis.

## Figures and Tables

**Table 1:** Tumor properties of the 25 patients with GEP-NETs.

Case no.	Age	Sex	NET type	Tumor grade	KI-67 index (%)	Type of therapy	Times of therapy
1	72	M	g-NET	3	80	Etoposide and cis-platinum	6
2	71	M	g-NET	3	80	Etoposide and cis-platinum	2
3	49	F	p-NET	2	20	Etoposide and cis-platinum	2
4	47	M	SI-NET	3	90	Etoposide and cis-platinum	4
5	28	F	r-NET	3	80	Etoposide and cis-platinum	2
6	45	M	SI-NET	3	70	Etoposide and cis-platinum	3
7	66	M	p-NET	3	80	Etoposide and cis-platinum	2
8	59	M	r-NET	2	15	Everolimus	3
9	49	F	r-NET	1	1	Everolimus	2
10	55	F	p-NET	2	10	Everolimus	4
11	39	M	SI-NET	1	<1	Octreotide acetate	6
12	66	M	p-NET	2	5	Octreotide acetate	2
13	59	M	r-NET	2	15	Octreotide acetate	3
14	40	M	p-NET	2	8	Octreotide acetate	4
15	42	F	SI-NET	2	7	Octreotide acetate	6
16	69	M	p-NET	2	1	Octreotide acetate	4
17	62	F	SI-NET	1	<1	Octreotide acetate	3
18	37	M	SI-NET	2	5	Octreotide acetate	3
19	71	M	g-NET	3	80	Xeloda and temozolomide	4
20	42	F	p-NET	3	50	Sunitinib	3
21	56	M	r-NET	2	5	Sunitinib	2
22	46	F	p-NET	2	10	Sunitinib	5
23	29	M	p-NET	2	15	Sunitinib	4
24	37	M	SI-NET	2	5	Sunitinib	5
25	36	F	p-NET	1	<1	Sunitinib	8

g-NET, gastric neuroendocrine tumors; p-NET, pancreatic neuroendocrine tumors; SI-NET, small intestinal pancreatic neuroendocrine tumors; r-NET, rectal pancreatic neuroendocrine tumors.

**Table 2:** CT evaluation of treatment response of the 25 patients with GEP-NETs.

Case no.	Changes (%)				RECIST 1.1 response	PFS (months)
	Density	Volume	Size	Density and volume		
1	1.68	287.33	43.75	289.01	PD	2.0
2	22.86	229.06	−11.55	251.92	SD	3.4
3	17.05	31.79	22.91	48.84	PD	—
4	14.89	25.28	−6.3	40.17	SD	4.3
5	17.77	−28.74	−39.28	−10.97	PR	6.2
6	−2.95	−88.68	−76.76	−91.63	PR	9.4
7	−31.67	296	193.75	264.33	PD	3.3
8	11	81.25	47.62	92.25	PD	3.0
9	10.99	5	−22.96	15.99	PR	5.7
10	0.97	9.89	−5.22	10.86	SD	4.8
11	−27.84	−74.55	−10.94	−102.39	SD	9.8
12	−4.7	131.25	97.06	126.55	PD	1.9
13	−16.48	57.86	16.9	41.38	SD	—
14	−13.13	43.48	−14.47	30.35	SD	5.7
15	12.87	152.91	17.7	165.78	SD	3.2
16	5.39	−27.53	−58.96	−22.14	PR	—
17	0.65	33.61	−10.68	34.26	SD	—
18	5.79	199.12	−5.73	204.91	SD	5.6
19	−30.16	−1.61	77.1	−31.77	PD	4.4
20	28	18.24	11.28	46.24	SD	2.4
21	1.46	13.73	−2.38	15.19	SD	4.9
22	−24.17	112.5	42.86	88.33	PD	1.8
23	1.69	34.67	2.13	36.36	SD	4.2
24	23.67	5.24	−6.69	28.91	SD	3.5
25	29.23	49.52	−18.43	78.75	SD	3.5

**Table 3:** Tumor response evaluation with changes in total tumor volume and mean volumetric tumor density after treatment.

Tumor response by TRCIST version 1.1	Total tumor volumes	Mean volumetric tumor density	Patient no.	Total
Tumor progression	↑(287.3%, 31.8%, 81.3%)	↑(1.7%, 17.1%, 11.0%)	3	7
	↑ (296.0%, 131.2%, 112.5%)	↓ (31.7%, 4.7%, 24.2%)	3	
	↓ (1.6%)	↓ (30.2%)	1	

Tumor nonprogression	↓(88.7%, 74.6%)	↓(3.0%, 27.8%)	2	18
	↑ (57.9%, 43.5%)	↓ (14.5%, 13.1%)	2	
	↓(28.7%, 27.5%)	↑(17.8%, 5.4%)	2	
	↑	↑	12	

## Data Availability

The data used to support the findings of this study are available from the corresponding author upon request.

## Abbreviations

GEP-NETs: Gastroenteropancreatic neuroendocrine tumors
RECIST: Response Evaluation Criteria In Solid Tumors
CgA: Chromogranin A
ROIs: Regions of interest
CR: Complete response
PR: Partial response
SD: Stable disease
CT: Computed tomography
HU: Hounsfield unit.

## Supplementary Materials

Supplementary Materials Supplementary video file shows a 3D visualization of the segmented tumors in one patient.
